# *Xenopus fraseri*: Mr. Fraser, where did your frog come from?

**DOI:** 10.1371/journal.pone.0220892

**Published:** 2019-09-11

**Authors:** Ben J. Evans, Marie-Theres Gansauge, Edward L. Stanley, Benjamin L. S. Furman, Caroline M. S. Cauret, Caleb Ofori-Boateng, Václav Gvoždík, Jeffrey W. Streicher, Eli Greenbaum, Richard C. Tinsley, Matthias Meyer, David C. Blackburn

**Affiliations:** 1 Department of Biology, McMaster University, Hamilton, ON, Canada; 2 Max Planck Institute for Evolutionary Anthropology, Deutscher Platz, Leipzig, Germany; 3 Florida Museum of Natural History, University of Florida, Gainesville, FL, United States of America; 4 Department of Zoology, Beaty Biodiversity Research Centre, University of British Columbia, Vancouver, British Columbia, Canada; 5 Forestry Research Institute of Ghana, Kumasi, Ghana; 6 Institute of Vertebrate Biology of the Czech Academy of Sciences, Czech Republic; 7 Department of Zoology, National Museum, Prague, Czech Republic; 8 Department of Life Sciences, The Natural History Museum, London, United Kingdom; 9 Department of Biological Sciences, University of Texas at El Paso, El Paso, United States of America; 10 School of Biological Sciences, University of Bristol, Bristol, United Kingdom; Leibniz-Institute of Freshwater Ecology and Inland Fisheries, GERMANY

## Abstract

A comprehensive, accurate, and revisable alpha taxonomy is crucial for biodiversity studies, but is challenging when data from reference specimens are difficult to collect or observe. However, recent technological advances can overcome some of these challenges. To illustrate this, we used modern approaches to tackle a centuries-old taxonomic enigma presented by Fraser’s Clawed Frog, *Xenopus fraseri*, including whether *X*. *fraseri* is different from other species, and if so, where it is situated geographically and phylogenetically. To facilitate these inferences, we used high-resolution techniques to examine morphological variation, and we generated and analyzed complete mitochondrial genome sequences from all *Xenopus* species, including >150-year-old type specimens. Our results demonstrate that *X*. *fraseri* is indeed distinct from other species, firmly place this species within a phylogenetic context, and identify its minimal geographic distribution in northern Ghana and northern Cameroon. These data also permit novel phylogenetic resolution into this intensively studied and biomedically important group. *Xenopus fraseri* was formerly thought to be a rainforest endemic placed alongside species in the *amieti* species group; in fact this species occurs in arid habitat on the borderlands of the Sahel, and is the smallest member of the *muelleri* species group. This study illustrates that the taxonomic enigma of Fraser’s frog was a combined consequence of sparse collection records, interspecies conservation and intraspecific polymorphism in external anatomy, and type specimens with unusual morphology.

## Introduction

Alpha taxonomy–the discovery and naming of species–is the linchpin of our catalog of biodiversity. Accurate species identification and diagnosis allows us to study change in populations, species, and communities, better understand how evolution occurs, and explore processes that drive diversification, extinction, and adaptation. Species names are generally assigned by the first publication to describe them, and rules exist for naming, revising, and synonymizing species names–for example as defined for animals by the International Code of Zoological Nomenclature (http://iczn.org/). One specimen, or a series of specimens, is designated to serve as a reference (a “type”) that defines a species. In principle, comparison to type specimens allows one to attribute non-type specimens to a named species, or alternatively, to justify the recognition of a new species.

Taxonomic ambiguities may arise when type material is lost or destroyed, when intraspecific variation is high, when different species have undifferentiated morphology, and when new categories of data are collected, such as nucleotide sequences, which are difficult to assay in type material. An example of one such ambiguity is the case of Fraser’s clawed frog, *Xenopus fraseri*. In 1852, two frog specimens collected by Louis Fraser (1819/20–1883; British zoologist and collector) were added to the catalog of the British Museum of Natural History (BMNH; now the Natural History Museum); in 1905, G. A. Boulenger designated these specimens to be syntypes of a new species, *Xenopus fraseri* [1]. The two type specimens of *X*. *fraseri* (probably males) are distinguished from all other *Xenopus* species by the combined presence of two morphological characters: (1) vomerine teeth, found only in these type specimens, *X*. *muelleri*, and *X*. *fischbergi*, and (2) prehallux claws, present in these type specimens but not in *X*. *muelleri* or *X*. *fischbergi*, and present in several other species that lack vomerine teeth (e.g., *X*. *allofraseri*, *X*. *parafraseri*) [2, 3]. The geographic origin of *X*. *fraseri* type specimens is listed in the BMNH catalog as “West Africa” and inferred to be Nigeria or “Fernando Po” (= Bioko Island, Equatorial Guinea) [1], or southern Benin or southwestern Nigeria [2]. The name *X*. *fraseri* was previously applied to populations now recognized as *X*. *allofraseri* and *X*. *parafraseri* (e.g., [3, 4]), but the combined presence of vomerine teeth and prehallux claws distinguish the syntypes of *X*. *fraseri* from these species [2]. A third formalin-preserved specimen that was collected in northwestern Ghana in 1975 (CAS 146198; male) was tentatively assigned to *X*. *fraseri* based on external morphology [2]. Thus, while distinctive molecular variation in the mitochondrial and nuclear genomes distinguishes all other species of African clawed frogs from one another (e.g., [2]), genetic data are lacking from *X*. *fraseri* because only two (or possibly three) specimens have been identified, and these are either old or formalin-preserved, and none has associated genetic samples. It thus remains unclear whether *X*. *fraseri* is in fact a distinctive species, or alternatively whether one of the more recently described species (e.g., [2]) is a synonym of *X*. *fraseri*. This latter possibility could arise, for example, if there is intraspecific polymorphism in the presence of either of the two morphological characters that distinguish *X*. *fraseri* types from all other species. Furthermore, if *X*. *fraseri* is distinct from other described species, both its phylogenetic position and geographic range remain unknown.

An increased understanding of *Xenopus* species diversity is important for several reasons. At the most basic level, an accurate inventory of species and their distributions allows us to monitor our planet’s biosphere, including how diversification and extinction vary over time and space. There are also several implications for our understanding of evolution by allopolyploidization, and by extension, the genomic dynamics of duplicate genes. Other than the diploid *X*. *tropicalis*, all species of *Xenopus* are allopolyploid, including 16 allotetraploid, seven allooctoploid, and four allododecaploid species [2]. These diverse species of African clawed frogs evolved by “regular” speciation, where one ancestor diverges into two descendants, and also by “allopolyploid” speciation, where two ancestral species merge into one allopolyploid descendant species [5]. Interestingly, allopolyploidization happened independently several times in this group, and phylogenetic analyses point to the existence of three diploid and three tetraploid species that (1) existed in the past, (2) contributed their genomes to extant tetraploid, octoploid, or dodecaploid species, and (3) do not have a known descendant with the same ploidy level as the pre-allopolyploidization ancestral species [2, 6–8]. These postulated species are the “lost ancestors” of *Xenopus* polyploids [2, 6–8], and any newly identified *Xenopus* species has the potential to be one of these lost ancestors. Discovery of one or more of these lost ancestral species could open up fascinating avenues of research that explore dynamics between each half (subgenome) of an allopolyploid genome, including mobility and suppression of transposable elements, Dobzhansky-Muller interactions, recombination, pseudogenization, subfunctionalization, and neofunctionalization (reviewed in [5]). Such studies would be catalyzed by currently available resources for *Xenopus* genomics, including two high quality genome sequences [9, 10] and powerful tools for gene editing (e. g., [11]).

Thus, to further understand the species status, phylogenetic placement, and geographic distribution of *X*. *fraseri*, we used sensitive techniques to capture and sequence almost complete mitochondrial genomes from both *X*. *fraseri* type specimens and the putative conspecific specimen. For comparative purposes, we also generated complete or almost complete mitochondrial DNA genomes from all other *Xenopus* species, and we analyzed external and internal morphology, including micro computed tomographic (μCT) scans of a *X*. *fraseri* type specimen, a putative conspecific, and populations of another *Xenopus* species (*X*. *fischbergi*, inferred to be closely related to *X*. *fraseri* by our phylogenetic analysis). We also followed up our phylogenetic inferences from complete mitochondrial DNA genomes, with Sanger sequencing of mitochondrial DNA from specimens we collected in the field. Our findings resolve the taxonomic quagmire presented by Mr. Fraser’s frog by establishing *X*. *fraseri* as a distinct species that occurs in northern Ghana and northern Cameroon, and is the sister taxon to *X*. *fischbergi*.

## Materials and methods

### Targeted high-throughput sequencing of mitochondrial genomes

We used targeted high-throughput sequencing to obtain complete or almost complete mitochondrial genome sequences from all *Xenopus* species, including both of the ~170-year-old type specimens of *X*. *fraseri*. We additionally obtained a partial mitochondrial genome sequence from the putative conspecific specimen of *X*. *fraseri* (CAS 146198) and an almost complete mitochondrial genome sequence from an unusual specimen of *Xenopus* (MZUF 16294) that was purportedly collected in Eritrea. We also used Sanger sequencing to generate partial mitochondrial sequences from recently collected specimens of *X*. *fischbergi* individuals from Chad and the Democratic Republic of the Congo, and *X*. *fraseri* individuals from northern Ghana and northern Cameroon. Apart from the putative conspecific specimen of *X*. *fraseri* (CAS 146198), the partial mitochondrial DNA sequences spanned portions of the cytochrome c oxidase I gene, the 12S and 16S ribosomal RNA genes, and the intervening tRNA^val^. Sample information is presented in S1 Table, and details of genetic data and capture probes, are provided in S1 Supporting Information.

#### Museum samples

Long-term storage of the four museum specimens studied here was in 60–70% ethanol or denatured alcohol, but the initial preservation treatments differed. Although we do not know what protocol was followed by Mr. Fraser when he collected the lectotype and the paralectotype specimens of *X*. *fraseri* (BMNH 1947.2.24.78 and BMNH 1947.2.24.79, formerly BMNH 1852.2.22.23 and BMNH 1852.2.22.24, respectively), it is likely that they were never exposed to formalin because the BMNH did not use formalin until the 1940s – 1950s (Colin McCarthy, personal communication). Liver and leg muscle were sampled from these specimens through small incisions made in the skin; the liver samples were used for targeted high-throughput sequencing. The putative conspecific specimen of *X*. *fraseri* (CAS 146198) was initially preserved in formalin; a sample of liver tissue from this specimen was used. A fourth museum specimen (MZUF 16294) was probably also initially preserved with formalin; a sample of muscle tissue from this specimen was used. The fourth museum specimen, a female, was listed as originating from Tessenei, Eritrea, and collected by M. Levrini in 1956. This specimen was included because the morphology was unusual for this locality because the specimen has claws on three toes of each hind foot, but the only known species in Eritrea–*X*. *clivii–*has four (including one on the prehallux). This specimen also served as a technical replicate for the performance of our targeted high-throughout sequencing on formalin preserved tissues.

### Assembly and inference of complete mitochondrial genomes

Reads were demultiplexed based on exact matches to sample-specific barcodes. Overlapping paired-end reads were then merged into consensus reads when possible. A *de novo* assembly of the complete mitochondrial genome was attempted for each sample with Trinity version 2.5.1 [12]. The advantage of a *de novo* assembly over a reference-based assembly is that the former approach may more accurately reconstruct insertion deletion polymorphisms, and presumably is less biased by a reference sequence.

The *de novo* assembly produced one ~17kb contig for the entire mitochondrial genome for almost all of the non-museum samples (28 out of 29), but none of the museum samples. For 5 of the 29 non-museum samples (*X*. *tropicalis*, *X*. *mellotropicalis*, *X*. *victorianus*, *X*. *laevis*, and *X*. *wittei*) the initial *de novo* assembly produced an assembly with two concatenated mitochondrial genomes. One sample (*X*. *poweri*) produced a fragmented assembly, and one of the two *X*. *boumbaensis* samples assembled only a small portion of the mitochondrial genome. For the five samples that initially produced concatenated mitochondrial genomes assembly, the *de novo* assembly was repeated using a k-mer size equal to either 21 or 32 instead of the initial setting of 29. For *X*. *victorianus* and *X*. *mellotropicalis*, a k-mer size equal to 21 provided a non-concatenated assembly, and the same for *X*. *laevis* and *X*. *wittei* with a k-mer size equal to 32. For *X*. *poweri*, the assembly was fragmented into 4 contigs that were independently aligned to the other *de novo* genomes with the others using Mafft [13] using the “—adjustdirection” option to align reverse-complemented contigs. The *X*. *poweri* sequence was then concatenated manually into a complete genome. For the *X*. *boumbaensis* sample that did not fully assemble *de novo*, we instead used the other *X*. *boumbaensis de novo* mitochondrial genome sequence as a reference, and generated a consensus sequence from reads that were mapped to this reference as described below for the museum samples.

Because *de novo* assembly of the museum samples failed to produce a complete mitochondrial genome, we instead generated a consensus sequence from unique and de-duplicated reads from each sample that were mapped to a reference mitochondrial genome. For each sample, we explored the effect of mapping to three different reference genomes that we generated *de novo* (*X*. *laevis*, *X*. *parafraseri*, *X*. *fischbergi*). We used as a reference the genome that yielded the most complete consensus, which was the *X*. *fischbergi* mitochondrial genome for the *X*. *fraseri* lectotype and paralectotype specimens, for the putative *X*. *fraseri* conspecific, and the *X*. *laevis* mitochondrial genome for the Eritrea specimen. Consensus calling on mapped reads requiring a coverage of at least 5x for each base, at least 80% agreement of the genotype, and a minimum map quality of 25.

### Phylogenetic analysis

To evaluate the phylogenetic position of the mitochondrial genome sequences from *X*. *fraseri*, we performed phylogenetic analysis on these sequences along with previously published mitochondrial genome sequences of several species in Pipoidea (the clade that contains the sister families Rhinophrynus and Pipidae) including *Rhinophrynus dorsalis* (HM991334.1), *Pipa carvalhoi* (HM991332.1) and *P*. *pipa* (GQ244477.1), *Hymenochirus boettgeri* (HM991331.1), and *Pseudhymenochirus merlini* (HM991333.1), *X*. cf. *tropicalis* (AP014695), *X*. *tropicalis* (AY789013), and *X*. *borealis* (JX155859). We excluded four regions from the alignment of these data where we deemed homology to be ambiguous based on visual inspection. These regions included the entire tRNA^Pro^ and D-loop (positions 15,500–17,610 of AY789013.1), two portions of the 16S rDNA gene (positions 1099–1140 and 2214–2228 of AY789013.1), and a portion of the origin for light strand replication (positions 5231–5248 of AY789013.1). To explore the effects of missing data, we performed separate phylogenetic analysis on an alignment that included only the complete or almost complete mitochondrial DNA genomes, and on an alignment that included these data plus the partial mitochondrial DNA genome sequences (S1 Table, S1 Supplemental File).

IQ-TREE version 1.6.8 [14, 15] was used for maximum likelihood phylogenetic analysis and model selection (the GTR+F+I+G4 model was selected for both datasets–with and without the partial mitochondrial sequences–according to the Bayesian Information Criterion), and ultrafast bootstrap analysis (with 1,000 replicates) was used to assess support for this topology. We also analyzed the both datasets with BEAST version 2.52 [16], using the same model of evolution listed above, a Yule model for the tree prior, and assuming an uncorrelated lognormal relaxed molecular clock. Three calibration points were used, all with a normally distributed prior probability distribution, and all following estimates in [17]: (1) the age of genus *Xenopus*, which subtends the subgenera *Silurana* and *Xenopus* [2], was set to a mean of 45.3 million years (my), and a standard deviation of 5.7 my, (2) the age of family Pipidae was set to a mean of 117.6 and a standard deviation of 6.6 my, and (3) the age of Pipoidea was set to a mean of 159.4 my and a standard deviation of 6.0 my. For each BEAST analyses, 4 or 8 independent runs were performed respectively, each for 10 million generations, sampling every thousand generations, and starting from a random tree. Twenty-five percent of each run was discarded as burn-in. Tracer version 1.71 [18] was used to verify that the effective sample sizes of all parameters exceeded 200.

### Morphology

We previously collected high-resolution X-ray μCT scans of several *Xenopus* specimens, including the paralectotype of *X*. *fraseri* and the holotype of *X*. *fischbergi* [2]. We used the same methodology to generate a scan of the putative *X*. *fraseri* specimen from Ghana (CAS 146198) and several more specimens of *X*. *fischbergi*, including one adult of each sex and two juveniles from Chad (specimens AMNH-H-A158343, AMNH-H-A158360, AMNH-H-A158350, and AMNH-H-A158356). These specimens were selected to capture variation between the sexes and during development. We lacked data from these post-metamorphic individuals from Chad, but inferred them to be *X*. *fischbergi* based on sequence from another individual (a tadpole) that was collected at the same time and localition (AMNH A-158377; S1 Table). We additionally examined external morphology and measured snout-vent lengths from several individuals for which we had sequence data to confirm species identification as *X*. *fraseri* or *X*. *fischbergi* (the sister taxon of *X*. *fraseri*, see below), and we also took measurements from the two adult *X*. *fischbergi* specimens from Chad.

### Ethics statement

All procedures involving live animals have been approved by the Animal Use Committee at McMaster University (AUP 17-12-43).

## Results

### *Xenopus fraseri* is distinct from all other species, the sister species of *X*. *fischbergi*, and minimally distributed in northern Ghana and northern Cameroon

**Fig 1:**
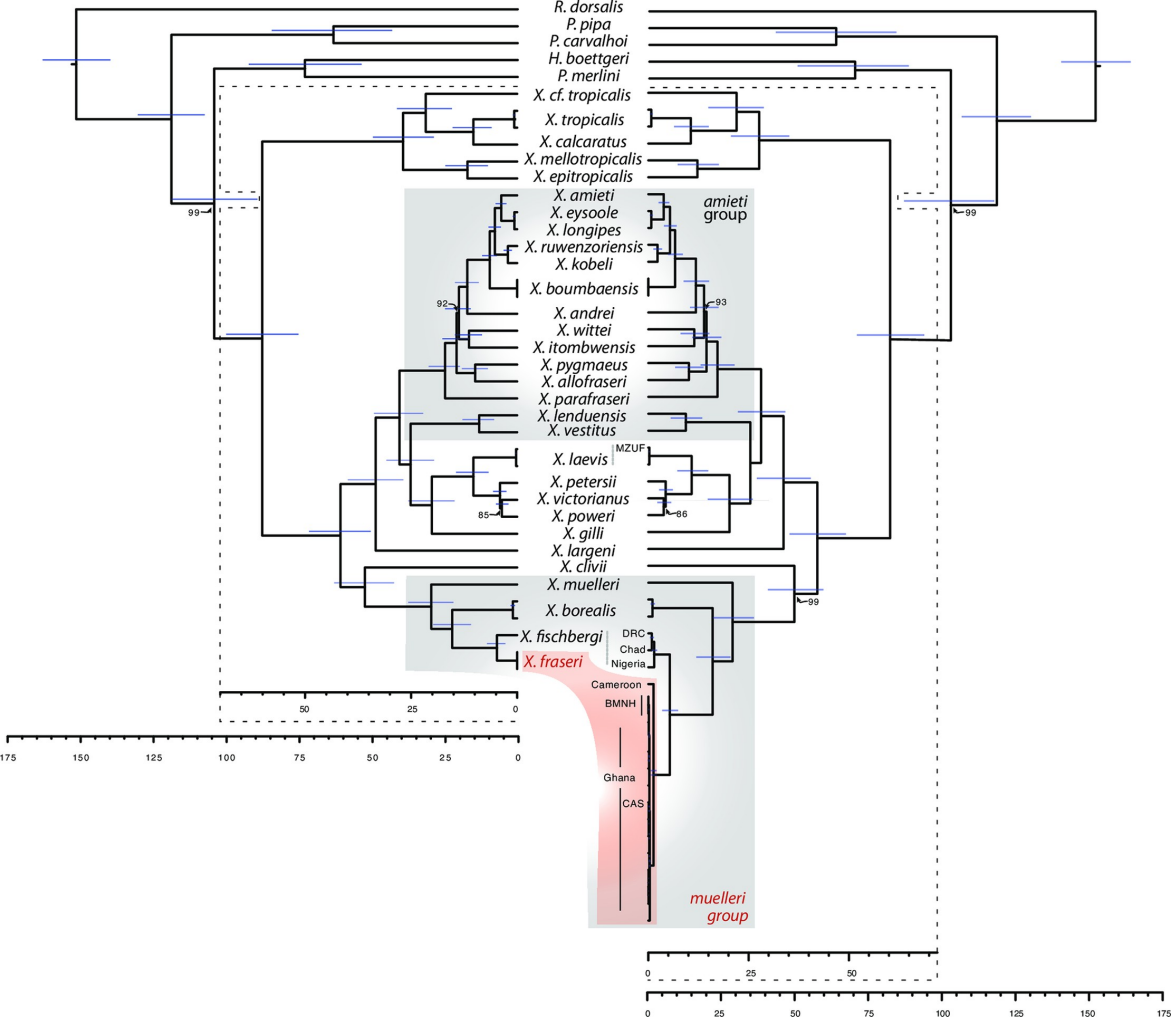
**Baysian phylogenetic analysis of *Xenopus* mitochondrial DNA complete or almost complete genomes (left), and these data plus partial mitochondrial sequences (right).** Two almost complete genomes from the lectotype and paralectotype of *X*. *fraseri* were included in both analyses; these genomes are labeled BMNH on the right and unlabeled on the left. All other *Xenopus* species have at least one complete or almost complete mitochondrial DNA genome. GenBank mitochondrial genomes from *X*. *borealis* and *X*. *tropicalis* were almost identical to ones generated, and the mitochondrial genome of specimen MZUF 16294 (MZUF), also unlabeled on the left, is almost identical to the mitochondrial genome we generated for *X*. *laevis*. The putative *X*. *fraseri* specimen CAS 146198 (CAS), type specimens of *X*. fraseri, and samples from Ghana are almost identical to one another. All nodes have 100% posterior probability except where indicated. Node bars indicate 95% highest posterior density for the node age. Scale bars are in millions of years.

Using targeted high-throughput sequencing, we obtained complete or almost complete mtDNA sequences from at least one representative of all *Xenopus* species, and including three of the four museum samples, and including both of the type specimens of *X*. *fraseri* (average length of all but CAS 146198 was 17,192 base pairs (bp), the range was 14,204–17,833 bp). A partial (5,930 bp) mitochondrial genome sequence was obtained from the more recently collected putative *X*. *fraseri* specimen from Ghana (CAS 146198). Phylogenetic analysis of these genomes indicates that mitochondrial DNA from both type specimens of *X*. *fraseri* are closely related, substantially diverged from other *Xenopus* species, and sister to mitochondrial sequences of *X*. *fischbergi* (Fig 1, S1 Fig).

Analysis of complete or almost complete mitochondrial DNA genomes were inferred by Bayesian and maximum likelihood phylogenetic methods identified no strongly supported topological differences, and most nodes had strong posterior probability and bootstrap support (Fig 1, S1 Fig). The BEAST analysis indicates that the mitochondrial genomes of *X*. *fraseri* and *X*. *fischbergi* diverged ~4.7 million years ago (mya; 95% highest probability density interval 2.8–7.1 mya).

These findings in mind, we then examined other wild-caught individuals sampled throughout the putative ranges of *X*. *fischbergi* and *X*. *fraseri* in five countries in West and Central Africa: the Democratic Republic of the Congo, Cameroon, Nigeria, Chad, and Ghana. Phylogenetic analysis of partial mitochondrial DNA Sanger sequences from these individuals combined with the mitochondrial genome sequences described above demonstrates that *X*. *fraseri* is distributed in northern Ghana and northern Cameroon, including CAS 146198, whereas *X*. *fischbergi* occurs in the Democratic Republic of the Congo, Chad, and Nigeria (Fig 1, S1 Fig). In both of these analyses (complete mitochondrial genomes with or without the partial sequences), the phylogenetic position of *X*. *fraseri* places it unambiguously within the *muelleri* species group, which includes as well *X*. *muelleri*, *X*. *borealis*, and *X*. *fischbergi* [2]. The relatively recent divergence of *X*. *fraseri* within the *muelleri* species group indicates that it is not one of the lost ancestors, and suggests that *X*. *fraseri* could be an allotetraploid species with 36 chromosomes, although we did not generate a karyotype to assess this.

### Divergence in mitochondrial DNA

Molecular divergence between the mtDNA sequences of the lectotype and paralectotype of *X*. *fraseri* was low: the uncorrected pairwise divergence (hereafter, divergence) was 0.11% out of 13,909 non-missing and non-gapped base pairs (hereafter, positions). Divergence between each of these sequences and the putative *X*. *fraseri* conspecific was also low: 0.22% out of 5,889 positions. But the complete genome of *X*. *fischbergi* from Nigeria was substantially diverged from almost complete mitochondrial genomes of the lectotype and paralectotype specimens of *X*. *fraseri* (4.37% or 4.53% out of 13,959 or 15,350 positions, respectively) and 2.7% diverged from the partial mitochondrial genome from the putative *X*. *fraseri* conspecific (out of 5889 positions). The smaller portions of the mitochondrial genome of *X*. *fischbergi* from Chad were also substantially diverged from the lectotype and paralectotype specimens of *X*. *fraseri* individuals (3.43% and 3.59% out of 2,796 or 2,842 positions, respectively) and from the partial mitochondrial genome from the putative *X*. *fraseri* conspecific (2.35% out of 2003 positions).

These divergences between *X*. *fraseri* and *X*. *fischbergi* exceed that between several other allotetraploid *Xenopus* sibling species pairs such as *Xenopus petersii* and *X*. *victorianus* (these two species are 3.43% diverged over their whole mitochondrial genomes, 1.87% over the portion of the genome that was sequenced for the putative *X*. *fraseri* conspecific, and 1.36% over the portion of the mitochondria that was sequenced for the *X*. *fischbergi* individual from Chad). Divergences between various dodecaploid species pairs (e.g., *X*. *kobeli* and *X*. *ruwenzoriensis*) are even lower than that observed between *X*. *petersii* and *X*. *victorianus* (Fig 1, S1 Fig). Divergence among mitochondrial DNA of *X*. *tropicalis* and *X*. cf. *tropicalis* is considerably larger than most other species owing to an unusual haplotype from Liberia [19, 20] that may constitute a distinct species [19] (with caveats based on one nuclear gene [2, 21]).

### Role of reference genome in consensus sequences

Although most of the mitochondrial genomes reported here were assembled *de novo*, the mitochondrial DNA genomes for the museum samples were generated by mapping capture data to a closely related reference genome. A possible concern with this approach is that the reference-based consensus sequences for the museum samples might be affected by which reference genome was used. To explore this, we compared the consensus sequences from each *X*. *fraseri* type specimen that were generated when either *X*. *fischbergi* or *X*. *laevis* was used. This comparison demonstrated that the consensus sequences were minimally biased, and far more complete when a closely related reference genome was used compared to a more distantly related reference genome (Supplemental Results). Our analyses suggest that the reference genome imposes only a modest bias on consensus sequences, but to the extent that it does, our figures for divergence between the mitochondrial genomes of *X*. *fraseri* and *X*. *fischbergi* may be slight underestimates.

### Comparative anatomy

**Fig 2:**
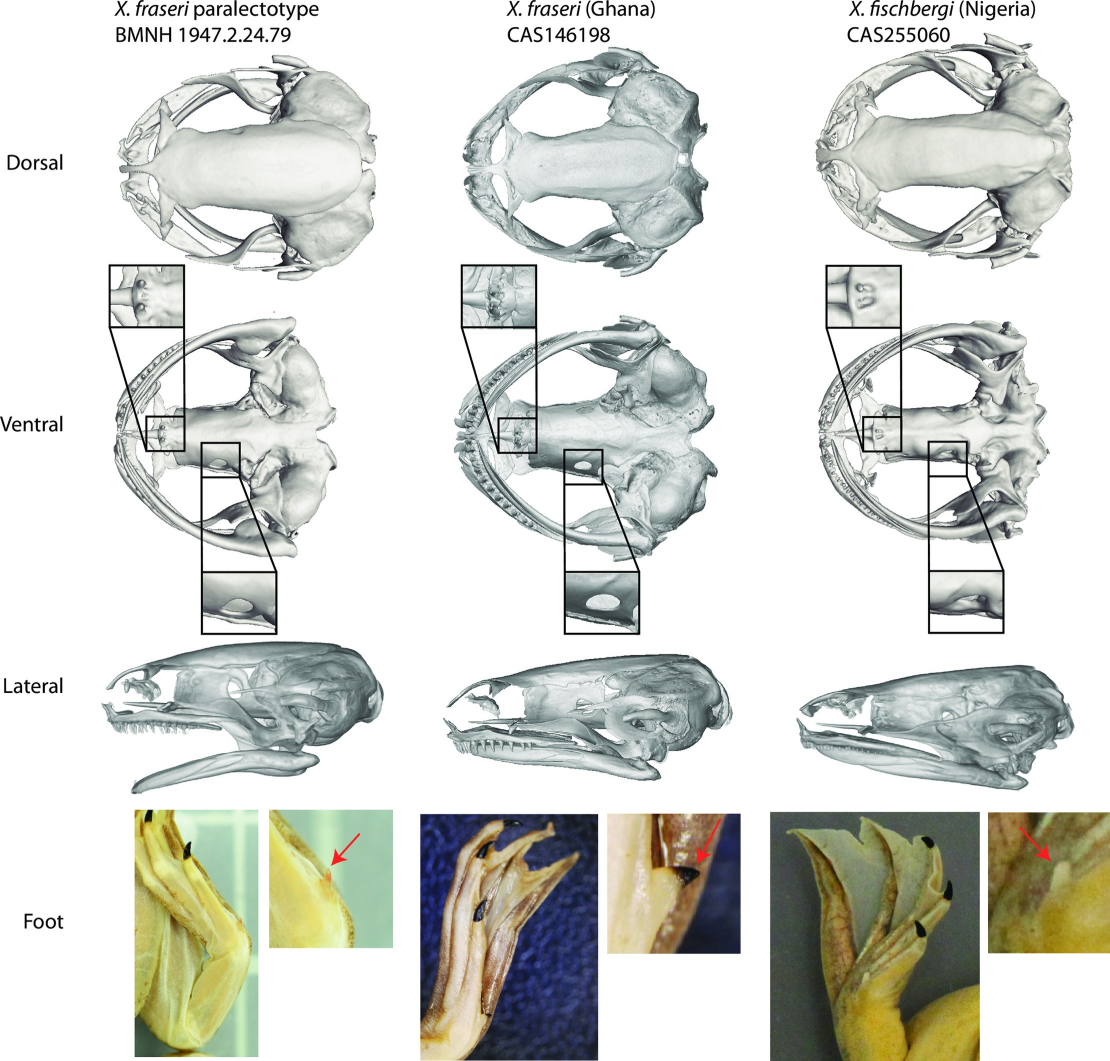
μCT scans and external morphology of the paralectotype of *X*. *fraseri* (BMNH 1947.2.24.79; probably male, SVL = 32.5 mm), a *X*. *fraseri* conspecific (CAS 146198; male, SVL = 34.1 mm), and the holotype of *X*. *fischbergi* (CAS 255060; female, SVL = 51.7 mm). On the ventral view, vomerine teeth and optic foramina are enlarged.

Comparison of external and internal anatomy of the putative *X*. *fraseri* conspecific CAS 146198 to the *X*. *fraseri* paralectotype (BMNH 1947.2.24.79) and the holotype of *X*. *fischbergi* (CAS 255060) confirms the presence of vomerine teeth (insets in Fig 2). A pointed keratinized prehallux claw is absent in *X*. *fischbergi* and appears to be an intraspecific polymorphism in *X*. *fraseri* that distinguishes some specimens of *X*. *fraseri* from specimens of other species (Supplementary Results; S2 Fig). The small size (for the *muelleri* species group [2]) of the lectotype and paralectotype of *X*. *fraseri* and the *X*. *fraseri* from Ghana (S2 Table) could suggest that they are not fully mature. However, examination of 13 other adult *X*. *fraseri* individuals from Ghana indicates that this species is indeed substantially smaller than *X*. *fischbergi*. The average SVLs of seven adult female and six adult male *X*. *fraseri* were 46.8 mm and 38.5 mm, respectively (ranges 39.9–54.2 and 36.9–32.5 mm respectively; S2 Table). The average SVL of the holotype and two paratypes (all female) of *X*. *fischbergi*, and two other female specimen from the Democratic Republic of the Congo (UTEP21194), is 57.8 mm (S2 Table), which is 25% larger than the *X*. *fraseri* females we measured. An adult male *X*. *fischbergi* we measured was larger than all seven males of *X*. *fraseri* that we measured (S2 Table).

Subtle differences in skeletal morphology are suggested by μCT scans. In the ventral view, the optic foramina of the *X*. *fischbergi* holotype and other specimens have more rounded margins on the fronto-parietal bone, especially in the adult specimens, compared to the *X*. *fraseri* paralectotype and putative conspecific (Fig 2, S3 Fig). However, the small sample of μCT scans prevents us from assessing whether these differences are distinctive in each species.

### Other phylogenetic insights

Our analysis of complete mitochondrial genome sequences from all *Xenopus* species provides strong statistical support for several novel or previously poorly supported relationships among mitochondrial DNA lineages in *Xenopus* (e.g., [2]), including strong support for (i) a sister relationship between *X*. *largeni* and a clade comprising mitochondrial lineages of species in the *laevis* and *amieti* species groups, and methodologically variable support (strong for Bayesian but modest for maximum likelihood) for (ii) a sister relationship between mitochondrial DNA from (*X*. *lenduensis* + *X*. *vestitus*) and species in the *laevis* species group, that renders mitochondrial DNA of the *amieti* group paraphyletic, and (iii) a sister relationship between *X*. *clivii* and species in the *muelleri* group (including *X*. *fraseri*). The complete mitochondrial genome from a museum specimen that was reportedly collected in Eritrea (MZUF 16294) was essentially identical to a complete mitochondrial genome from *X*. *laevis* from South Africa. This suggests that this specimen either was introduced to Eritrea from southern Africa where *X*. *laevis* occurs, or (we suspect) was mislabeled, and actually originated from South Africa. This specimen indicates that formalin preserved material has the potential to yield complete mitochondrial DNA genomes, even though we did not obtain one from CAS 146198.

## Discussion

Although we do not advocate using divergence of mitochondrial DNA as the sole justification for species distinctiveness, these data coupled with morphological characteristics discussed above, including the substantially smaller size of *X*. *fraseri* compared to other closely related species (S2 Table, [2]), and a pointed prehallux in some *X*. *fraseri* individuals, argues for the distinctiveness of *X*. *fraseri*. The specimens of *X*. *fraseri* were sampled from 4 different localities, and internal examination of one female indicated that she was gravid. This suggests the smaller size of these individuals compared to *X*. *fischbergi* is unlikely to be due to ontogeny. Our findings indicate that the sister taxon of *X*. *fraseri* is *X*. *fischbergi*, and that *X*. *fraseri* is a member of the muelleri species group along with *X*. *muelleri*, *X*. *fischbergi*, and *X*. *borealis* (Fig 1, S1 Fig). The minimal distribution of *X*. *fraseri*, established in this study, is in northern Ghana and Cameroon. *Xenopus fraseri* may be partially sympatric with *X*. *fischbergi*, the latter of which ranges at least from Nigeria to the northern Democratic Republic of the Congo (based on mitochondrial sequences), and possibly even farther west and east of this [2]. Given the similarity in external morphology of *X*. *fraseri* and *X*. *fischbergi*, our previous delineation of the range of *X*. *fischbergi* [2] may be an overestimate and, based on genetic information, we reassign several paratypes of *X*. *fischbergi* to *X*. *fraseri*, as detailed in the Supplemental Results.

Our recent systematic revision of West African *Xenopus* [2] included sequence data from two genetic samples that were identified here to be *X*. *fraseri*, but we failed to identify them then as being distinctive from *X*. *fischbergi* [2]. At that time, we did not realize that the shape and morphology of the prehallux was so variable within *X*. *fraseri*. The prehalluxes of the *X*. *fraseri* types and the formalin-preserved specimen from Ghana (CAS 146198) are the most keratinized specimens we have encountered (S2 Fig), so the type specimens of *X*. *fraseri* are in fact not particularly “typical” of this species in this regard. Additionally, because the mitochondrial DNA data in [2] was from a relatively slowly evolving portion of this genome, the divergence between the *X*. *fraseri* and *X*. *fischbergi* data in [2] was lower (~3%) than that between the almost complete mitochondrial genomes that were generated for this study (>4%; Supplemental Results).

In species with low dispersal rates, divergent and geographically separated intra-specific clades with narrow regions of overlap may arise without geographic barriers to gene flow [2]; this is seen, for example, in *X*. *laevis* from southwest South Africa [22]. In contrast, it is rare in species with low dispersal rates–including in *Xenopus* [23]–for diverged intra-specific clades of mitochondrial DNA to have broadly overlapping geographic distributions. This phylogeographic pattern is instead suggestive of cryptic species [2, 23], and the new genetic and morphological information from *X*. *fraseri* presented here allowed us to distinguish this cryptic species from another closely related, morphologically similar, and possibly co-distributed or parapatric species (*X*. *fischbergi*).

### Other phylogenetic insights

The inference of a closer phylogenetic affinity of the mitochondrial genome of *X*. *clivii* to the mitochondrial genomes of the *muelleri* species group than to those of the *laevis* or *amieti* species groups is in line with inferences based on complete transcriptomes [24]. This observation is of interest for studies of sex chromosomes because *X*. *clivii* and several species in the *laevis* and *amieti* species group carry the female-specific sex determining gene *dm-w* [25], whereas *X*. *borealis* does not carry *dm-w*, and has a newly evolved sex chromosome [26, 27]. This phylogenetic relationship of mitochondrial DNA, which is (also) maternally inherited, indicates that *dm-w* was lost at some point in the ancestry of *X*. *borealis* after the divergence of *X*. *clivii* [25].

### Conclusions

Because he died over a century ago, Mr. Fraser is unavailable to tell us exactly where he collected his frog. Despite the uncertainty over the origin of Mr. Fraser’s specimens, it has long been assumed that *X*. *fraseri* is distributed in lowland tropical forest [25]. This was reinforced by the many records of morphologically similar forest-dwelling specimens, including those now called *X*. *parafraseri* and *X*. *allofraseri* [28]. In fact, *X*. *fraseri* is distributed in relatively hot, arid savanna, including the borders of the Sahel. We argued previously based on historical records that the lectotype and paralectotype specimens of *X*. *fraseri* were probably collected in southern Benin or southwestern Nigeria [2] in areas that are in or near the Dahomey Gap, an area of savanna extending south from the Sahel that subdivides tropical rainforest habitat of West Africa, and that is poorly studied for *Xenopus*. Consistent with this proposal, the localities of this species identified here–northern Cameroon and northern Ghana–are in the same ecoregions as the Dahomey Gap (West Sudanian savanna and Guinean forest-savanna mosaic) [2]. Previous confusion about *X*. *fraseri* arose as a consequence of poor records of the provenance of the type specimens, the generally conserved interspecific morphology of several *Xenopus* species, high-intraspecific polymorphism of the prehallux of *X*. *fraseri*, and the unusual prehallux morphology of the *X*. *fraseri* type specimens. This study thus provides clarity to this taxonomic enigma presented by Fraser’s frog by identifying distinctive molecular and morphological features of this species–as well as polymorphic aspects, and by delineating its phylogenetic position and geographic distribution.

## Supporting information

S1 Supporting Information Supplemental methods and results for this study. (DOCX)

S1 Table Species, sample and museum identification numbers, origin, and sex of genetic samples analyzed in this study. (XLSX)

S2 Table Snout-vent length of *X*. *fraseri* and *X*. *fischbergi* specimens (Voucher ID), including type and non-type specimens (Type Status) that are male or female (Sex). (XLSX)

S1 Fig **Maximum likelihood bootstrap consensus tree of *Xenopus* mitochondrial DNA complete or almost complete genomes (left), and these data plus partial mitochondrial sequences (right).** Bootstrap support is 100 percent except where indicated.
(TIF)

S2 Fig Prehallux morphology of *X*. *fraseri*. Some *X*. *fraseri* individuals have a rounded prehallux (left two images), a slightly pointed prehallux (center), or a pointed and keratinized prehallux (right two images). Thus, a pointed and/or keratinized prehallux is a distinguishing, but not universal, characteristic of *X*. *fraseri*.
(TIF)

S3 Fig μCT scans of four *X*. *fischbergi* specimens from Chad. On the ventral view, vomerine teeth and optic foramina are highlighted as in Fig 2. In the two juvenile specimens (right), one or both vomerine teeth are not X-ray opaque and are not visible on these scans, but they are present.
(TIF)

S1 Supplemental File Alignment of mitochondrial genome sequences analyzed in this study in nexus format. (NEXUS)
