# Supplementary material for: Xenopus fraseri: Mr. Fraser, where did your frog come from?
Source: PLoS One. 2019 Sep 11;14(9):e0220892. doi: 10.1371/journal.pone.0220892 (PMC6738922; doi:10.1371/journal.pone.0220892)
Supplement: S1 Supporting Information — (DOCX) [file pone.0220892.s001.docx]

**Supporting Information**

**Supplemental Methods**

**Targeted high-throughput sequencing**

DNA was extracted from all samples (including the museum specimens) using Qiagen DNEasy extraction kits, except the *X. gilli* sample where a phenol/chloroform extraction was performed. For the museum samples, single-strand sequencing libraries with sample-specific barcodes were constructed as described in [[1](#_ENREF_1), [2](#_ENREF_2)]. For the other samples, double-stranded libraries with sample-specific seven base pair (bp) barcodes were constructed as described in [[3](#_ENREF_3)].

Mitochondrial capture was performed using probes that were designed from four complete mitochondrial genome sequences (*X. tropicalis* GenBank accession AY789013.1, *X. laevis*, accession M10217.1, *X. victorianus*, accession JX155858.1, *X. borealis* accession JX155859.1), five partial mitochondrial DNA sequences spanning portions of the 12S and 16S ribosomal RNA genes and the intervening tRNA^val^ (*X. parafraseri* accession AY581631.1, *X. allofraseri* accession AY581632.1, *X. wittei* accession HQ225701.1, *X. ruwenzoriensis* accession AY581624.1, *X. clivii* accession AY581637.1), and seven partial mitochondrial DNA sequences spanning a portion of the cytochrome c oxidase I gene (*X. epitropicalis* accession EU566848.1, *X. clivii* accession EU566840.1, *X. allofraseri* accession EU566845.1, *X. lenduensis* accession HQ225705.1, *X. parafraseri* accession KT728011.1, *X. muelleri* accession EU599031.1, *X. fischbergi* accession EU588990.1). A perl script was used to generate all possible unique overlapping 52-bp probes with 1 base pair tiling from a multifasta file containing these data. An 8-bp flanking sequence was attached to the 3’ end of each of 80,230 unique probe sequences, and the resulting 60-bp probes were printed (with redundancy) on a one million feature probe array (Agilent, USA) as described previously in the supplement of [[2](#_ENREF_2)]. Capture probes were synthesized and used to hybridize to and pull out molecules in individual DNA libraries for sequencing, as described previously [[4](#_ENREF_4)]. Paired-end sequencing was performed on captured single and double stranded libraries on a portion of different lanes of an Illumina HiSeq 2500 machine.

**Supplemental Results**

**Impact of reference genome on consensus sequences**

To explore possible biases associated with the reference genome used to generate consensus sequences for mitochondrial genomes from museum specimens, we compared the consensus sequences from each *X. fraseri* type specimen that were generated when either the *X. fischbergi* or *X. laevis* mitochondrial genome was used as a reference genome. The consensus sequence was far more complete when the *X. fischbergi* reference was used (14,204 and 15,709 bp for BMNH 1947.2.24.78 and BMNH 1947.2.24.79, respectively) than when the *X. laevis* reference was used (5,112 and 5,050 bp, respectively). After excluding regions with missing genotype calls, the consensus sequences from the same individual that were generated from these two different reference genomes were almost identical (there was 1.02% divergence between the two 1947.2.24.78 consensus sequences and 0.04% divergence between the two BMNH 1947.2.24.79 consensus sequences). The differences between the two consensus sequences for the same individual always matched the *X. laevis* reference for consensus sequences that were made from the *X. laevis* reference. However, several within-individual differences between the two consensus sequences did not match the *X. fischbergi* reference in these consensus sequences that were made from the *X. fischbergi* reference (8 of 52 divergent sites in the 1947.2.24.78 consensus sequences from the *X. fischbergi* reference and 1 of 2 divergent sites in BMNH 1947.2.24.79 consensus sequences). These results suggest that the influence of the reference genome on the consensus genotypes was relatively small. It also indicates that using a closely related reference genome allowed us to get a more complete consensus sequence that better captured unique variation that was not found in the reference genome.

**The prehallux of *X. fraseri***

In addition to five *X. fischbergi* and two *X. fraseri* specimens that were used for µCT scans (sample identifications listed in the Results section), we inspected the external anatomy of one *X. fischbergi* (Democratic Republic of the Congo: UTEP21194) specimen, and 16 *X. fraseri* specimens (S1 and S2 Tables). We confirmed the species identification of these specimens using mitochondrial DNA sequences of the same individual, or for the specimens from Chad, another individual from the same population. This demonstrated that the pointed prehallux that is present in the type specimens (Fig. 2) is a distinguishing, but not universal, characteristic of *X. fraseri* (S2 Fig.).

**Reassignment of paratypes of *X. fischbergi* to *X. fraseri***

Our molecular analyses demonstrate that one of the paratype specimens of *X. fischbergi* is in fact *X. fraseri*: UWBM5964 (field ID ADL3542). Based on their collection locality in Ghana, we suspect as well that *X. fischbergi* paratypes BMNH 1983.1501-1520 are also *X. fraseri,* although we do not have sequence data from these specimens to confirm this. Additionally, molecular data analyzed herein indicates that at least one and probably both referred specimens from Djingliya, Cameroon, NMP6V 74744/1-2 (field ID VG 10-280 and VG 10-281 respectively), are *X. fraseri* rather than *X. fischbergi*.

**Supplemental References**

1. Miller CS, Gosling WD. Quaternary forest associations in lowland tropical West Africa. Quaternary Science Reviews. 2014;84:7-25. doi: 10.1016/j.quascirev.2013.10.027. PubMed PMID: WOS:000331682200002.

2. Gansauge MT, Gerber T, Glocke I, Korlevic P, Lippik L, Nagel S, et al. Single-stranded DNA library preparation from highly degraded DNA using T4 DNA ligase. NAR. 2017;45(10). doi: ARTN e79

10.1093/nar/gkx033. PubMed PMID: WOS:000402510700004.

3. Meyer M, Kircher M. Illumina sequencing library preparation for highly multiplexed target capture and sequencing. Cold Spring Harb Protoc. 2010;2010(6):pdb prot5448. doi: 10.1101/pdb.prot5448. PubMed PMID: 20516186.

4. Fu Q, Meyer M, Gao X, Stenzel U, Burbano HA, Kelso J, et al. DNA analysis of an early modern human from Tianyuan Cave, China. PNAS. 2013;110(6):2223-7. doi: 10.1073/pnas.1221359110. PubMed PMID: 23341637; PubMed Central PMCID: PMCPMC3568306.
