# Supplementary figures and images for: Xenopus fraseri: Mr. Fraser, where did your frog come from?
Source: PLoS One. 2019 Sep 11;14(9):e0220892. doi: 10.1371/journal.pone.0220892 (PMC6738922; doi:10.1371/journal.pone.0220892)

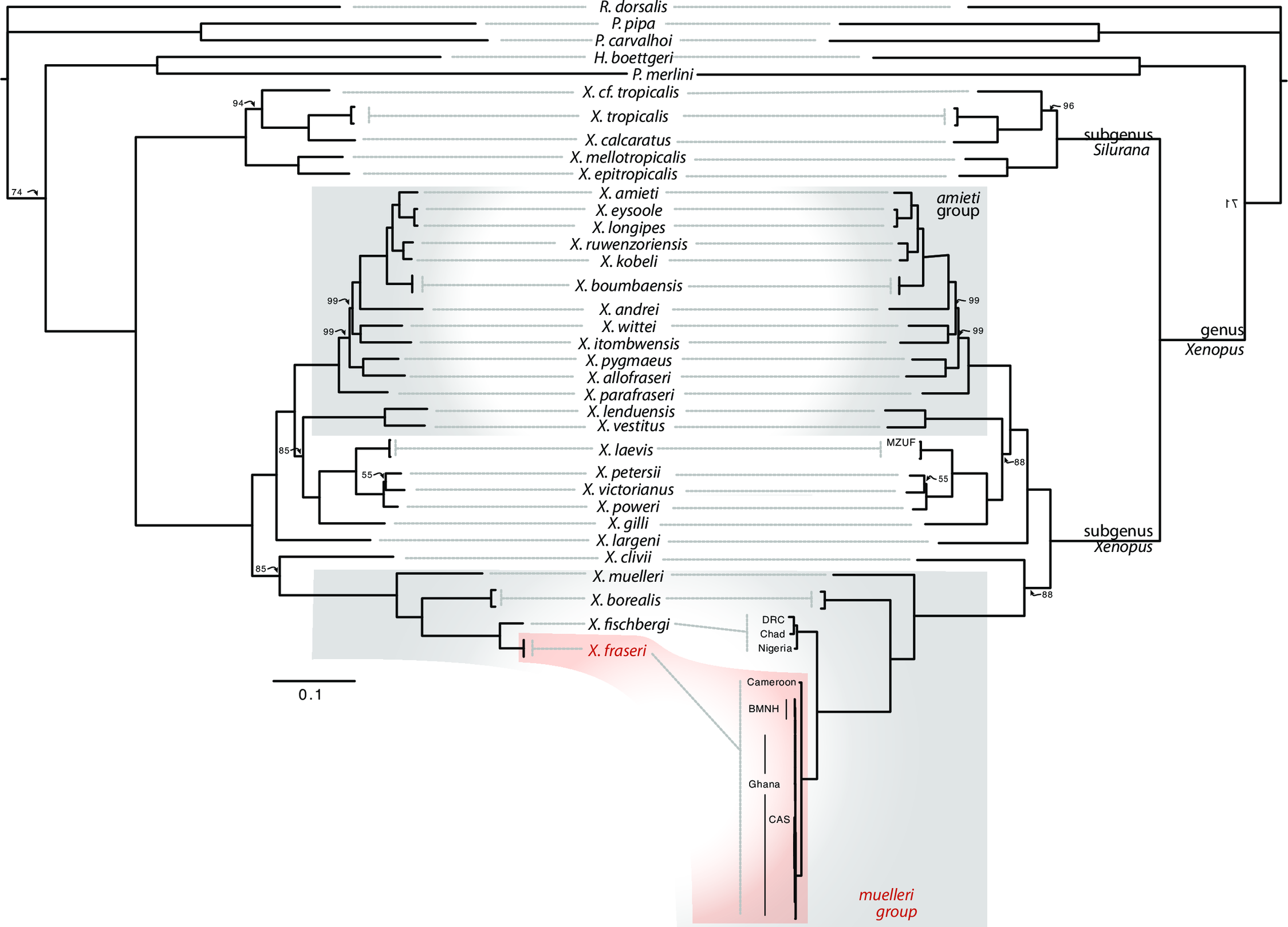

Supplement: S1 Fig — Maximum likelihood bootstrap consensus tree of Xenopus mitochondrial DNA complete or almost complete genomes (left), and these data plus partial mitochondrial sequences (right). Bootstrap support is 100 percent except where indicated. (TIF) [file pone.0220892.s004.tif]

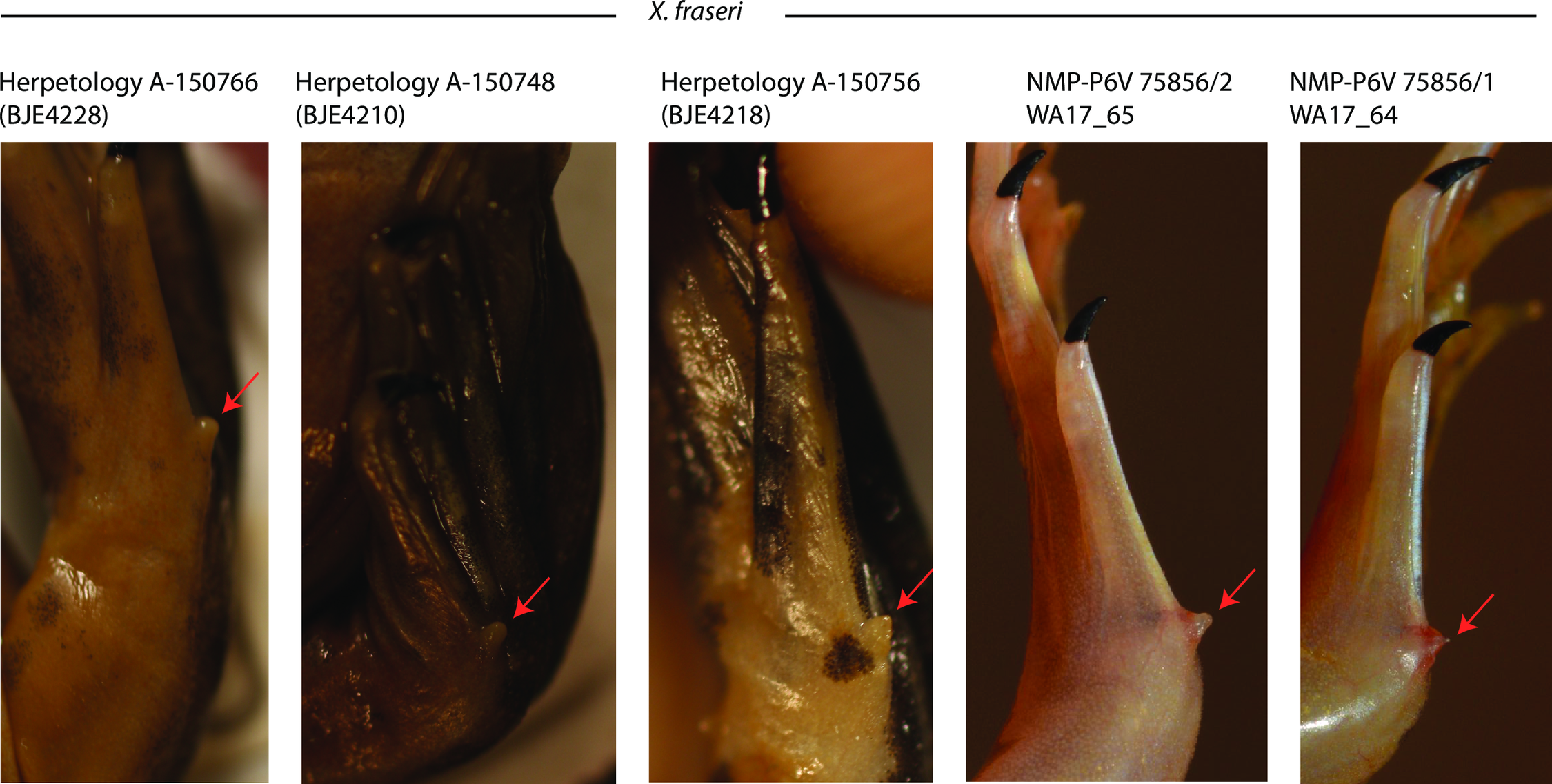

Supplement: S2 Fig — Some X. fraseri individuals have a rounded prehallux (left two images), a slightly pointed prehallux (center), or a pointed and keratinized prehallux (right two images). Thus, a pointed and/or keratinized prehallux is a distinguishing, but not universal, characteristic of X. fraseri. (TIF) [file pone.0220892.s005.tif]

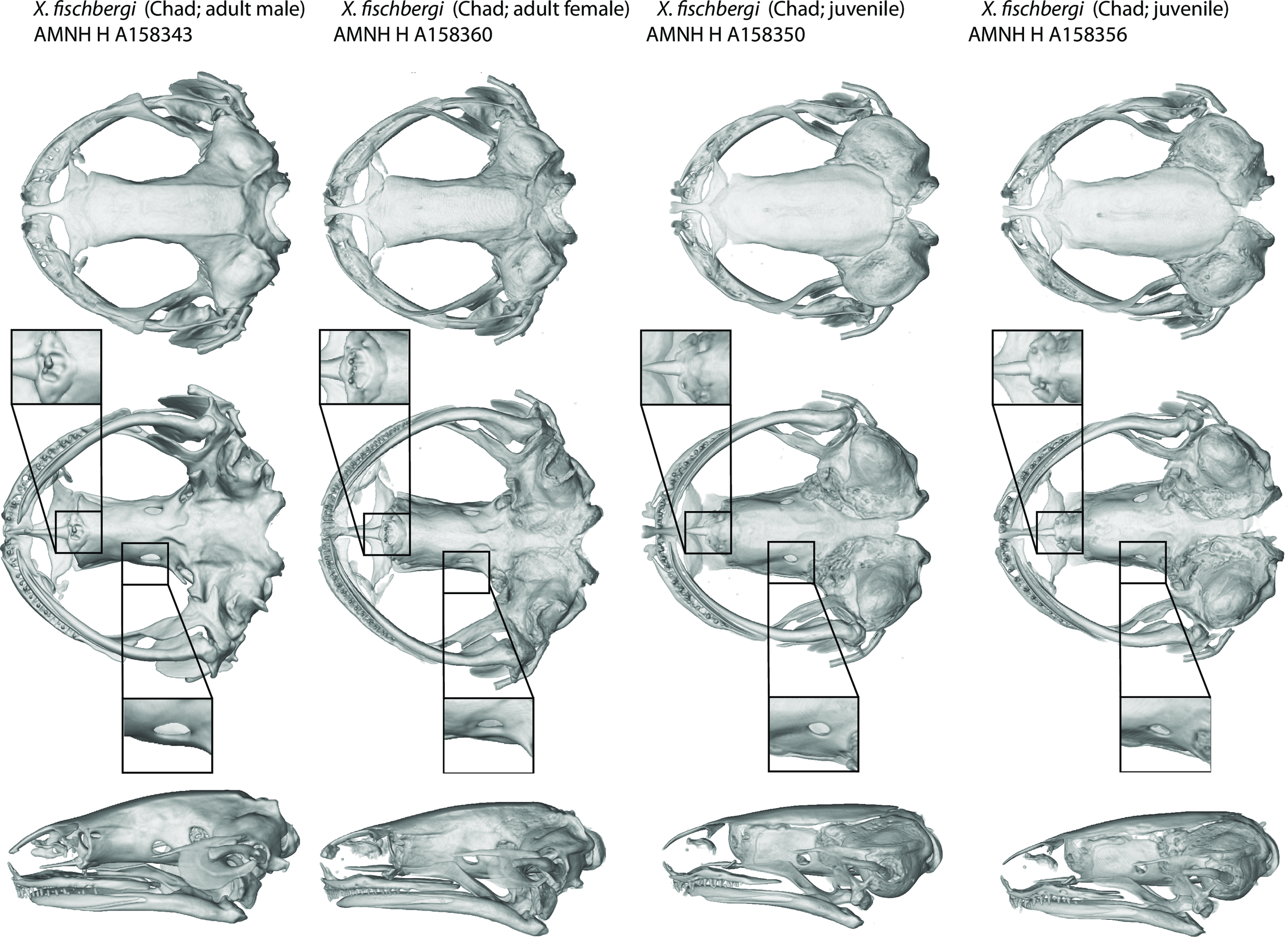

Supplement: S3 Fig — On the ventral view, vomerine teeth and optic foramina are highlighted as in Fig 2. In the two juvenile specimens (right), one or both vomerine teeth are not X-ray opaque and are not visible on these scans, but they are present. (TIF) [file pone.0220892.s006.tif]
